# Supplementary material for: Development of Anti-OSCAR Antibodies for the Treatment of Osteoarthritis
Source: Biomedicines. 2023 Oct 19;11(10):2844. doi: 10.3390/biomedicines11102844 (PMC10604876; doi:10.3390/biomedicines11102844)
Supplement: Supplementary file 1 [file biomedicines-11-02844-s001.zip › biomedicines-2629354-supplementary.pdf]

Supplemental Figures.

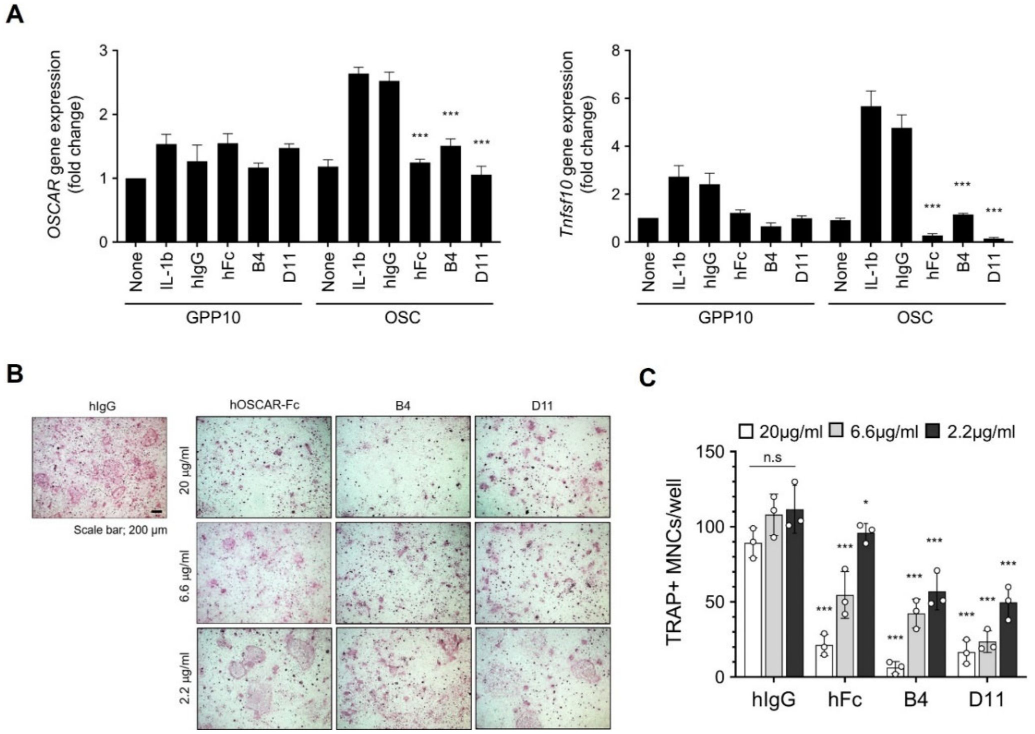

Figure S1. The B4 and D11 antibodies inhibit OSCAR activity in vitro.

(A) Primary articular chondrocyte assay. Primary articular chondrocytes were cultured with OSC or the GPP10 control peptide plus candidate antibodies, the positive control hOSCAR-Fc (10 μg/ml), or the negative control hIgG1 (10 μg/ml), for 2 h. IL-1β (5 ng/ml) was then added for 48 h. OSCAR and TRAIL mRNA levels were determined by quantitative RT-PCR. One-way ANOVA was performed followed by Dunnett's multiple comparisons test in GPP10 or OSC treated group. (B, C) Osteoclast-differentiation assay. Bone marrow-derived macrophages were co-cultured for 6 days with primary osteoblasts together with 1 μM prostaglandin E2, 10 nM 1,25-$(OH)_2$ vitamin D3, and various concentrations of B4, D11, or hOSCAR-Fc fusion protein. hIgG1 served as the negative control. The cells were then fixed and stained for TRAP activity (B) and the cells with more than three nuclei (multinucleated cells; MNC) were counted and presented in histograms (C). Error bars represent mean ± S.E.M. * $p < 0.05$, ** $p < 0.01$, and *** $p < 0.001$ when comparing the hOSCAR-Fc or anti-OSCAR Ab-treated groups with the hIgG-treated group at each concentration.

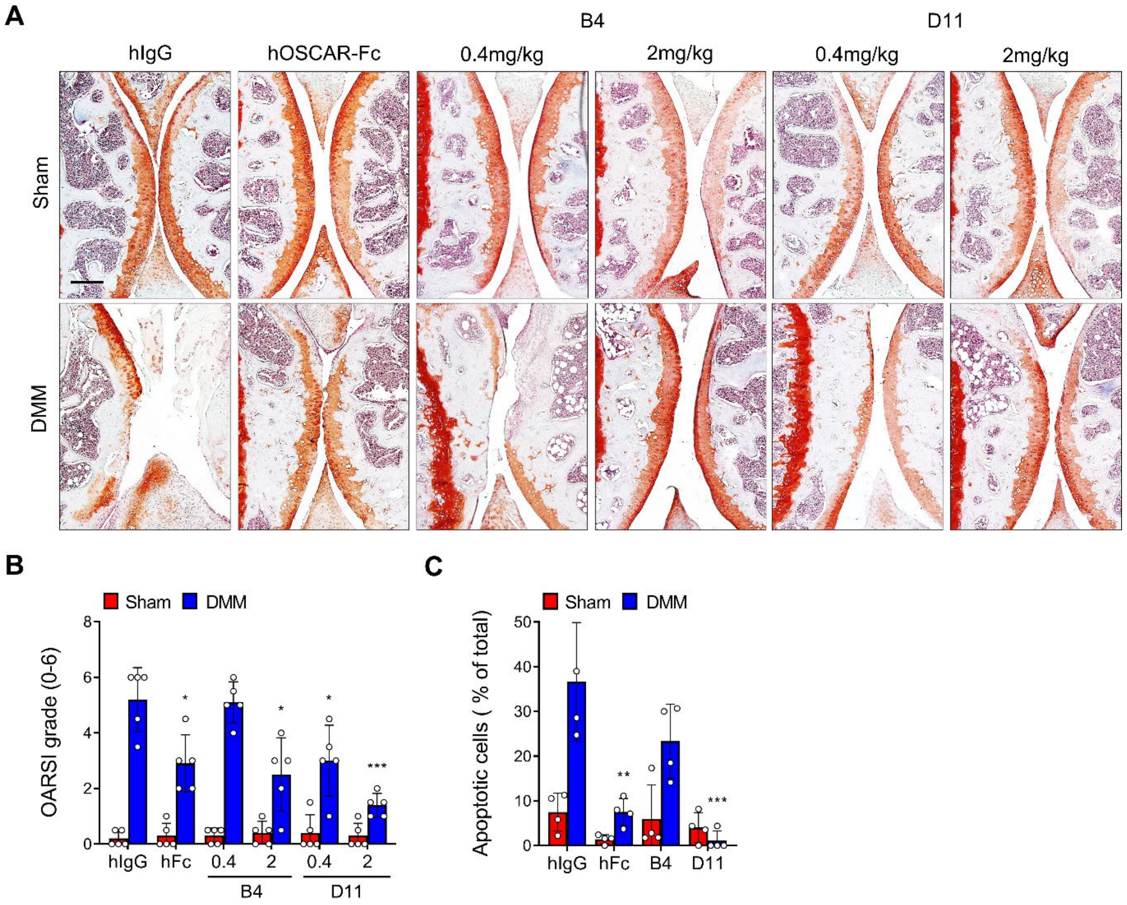

Figure S2. B4 and D11 attenuate the cartilage destruction and chondrocyte apoptosis in mice with DMM surgery-induced OA.

C57BL/6 male mice were subjected to sham or DMM surgery and, starting 1 week after surgery, given twice-weekly intra-articular injections of hOSCAR-Fc (2 mg/kg), B4 (0.4 mg/kg or 2 mg/kg), D11 (0.4 mg/kg or 2 mg/kg), or the isotype-matched hIgG1 control antibody for 8 weeks (n = 5 per group). The mice were sacrificed at 9 weeks. (A, B) Articular cartilage sections were subjected to safranin-O staining. Representative images are shown in (A). Scale bar: 100 μm. The OARSI scores were presented as a bar graph (B). (C) Apoptotic articular chondrocytes in the sections were detected and quantified by TUNEL assay. Error bars represent mean ± S.E.M. Two-way ANOVA followed by Sidak's Multiple Comparison test was conducted. * $p < 0.05$, ** $p < 0.01$ *** $p < 0.001$ when the hOSCAR-Fc-, B4-, or D11-treated groups were compared with the hIgG control group. hFc: hOSCAR-Fc.

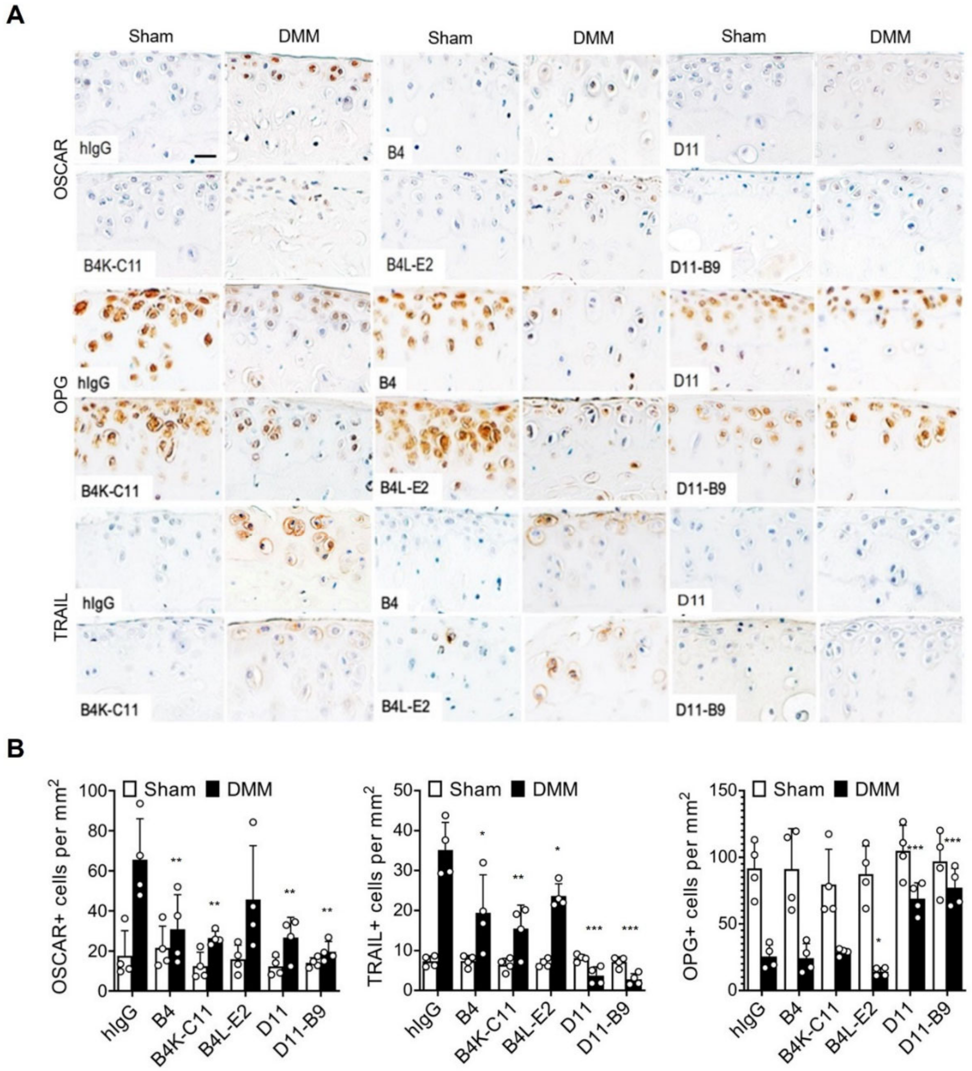

Figure S3. The anti-OSCAR B4 and D11 variant antibodies ameliorate OA and suppress TRAIL-induced articular chondrocyte apoptosis.

Mice underwent DMM or sham surgery and then twice-weekly intra-articular injections with B4, D11, or their affinity-matured variants for 8 weeks, starting the week after surgery (n = 6 per group). The mice were sacrificed 9 weeks after surgery. The articular cartilage tissues were subjected to immunohistochemical analysis of OSCAR, TRAIL, and OPG expression. (A) Representative images. Scale bar: 25 μm. (B) Quantitative analysis of the immunohistochemistry results. Error bars represent mean ± S.E.M. Two-way ANOVA followed by Sidak's Multiple Comparison test was conducted. * $p < 0.05$, ** $p < 0.01$, *** $p < 0.001$ when the anti-OSCAR antibody-treated groups were compared with the hIgG control group.
